# Association between atherogenic index of plasma and depression risk: a meta-analysis

**DOI:** 10.3389/fpsyt.2025.1665118

**Published:** 2025-11-19

**Authors:** Gui-Ling Chen, Gui-Xin Chen, Wei-Tao Wan

**Affiliations:** 1 Department of Psychiatry, Tianyou Hospital Affiliated to Wuhan University of Science & Technology, Wuhan, China; 2 Department of Neurology, Wuhan Third Hospital-Tongren Hospital of Wuhan University, Wuhan, China

**Keywords:** atherogenic index of plasma, depression, meta-analysis, lipid metabolism disorders, biomarker

## Abstract

**Background:**

Lipid metabolism disorders have been implicated in the pathogenesis of depression. The atherogenic index of plasma (AIP), calculated as log(triglyerides/high-density lipoprotein cholesterol), is a convenient marker reflecting lipid profiles and cardiovascular risk. However, the relationship between the AIP and depression remains unclear.

**Methods:**

Relevant observational studies were identified through comprehensive searches of the PubMed, EMBASE, and Web of Science databases. Studies were included if they reported AIP values and diagnosed depression using standardized assessment tools. A total of 10 observational studies, encompassing 38,785 participants, were included. Subgroup analyses were conducted to assess the impact of age and diagnostic criteria on the association. Heterogeneity was assessed using the I² statistic, and publication bias was evaluated using funnel plots and Egger’s test.

**Results:**

Individuals with depression had significantly higher AIP values compared with healthy controls (mean difference = 0.07; 95% confidence interval: 0.03–0.11; *P* = 0.0006). High heterogeneity was observed (*I²* = 94%, *P* < 0.00001). The funnel plot showed slight asymmetry; however, Egger’s test indicated no significant publication bias (*P* = 0.354). Sensitivity analyses confirmed the robustness of the findings.

**Conclusion:**

Higher AIP values are associated with an increased risk of depression, particularly in individuals aged ≥50 years. Given its accessibility, the AIP may serve as a useful biomarker for the early identification of individuals at risk for depression. The quantification of the overall association between AIP and depression risk represents a novelty of this study and highlights AIP as an integrative lipid biomarker with potential predictive value beyond single lipid indices. Prospective studies are needed to confirm causality and explore the underlying biological mechanisms.

**Systematic review registration:**

https://www.crd.york.ac.uk/prospero/, identifier CRD420251035701.

## Introduction

Depression is a major global public health issue, significantly contributing to disability, reduced quality of life, increased risk of comorbidities, and higher mortality rates (1, 2). Current estimates indicate that more than 280 million individuals worldwide are affected by this condition, highlighting its critical importance in both clinical practice and public health policy (3, 4). Although substantial progress has been made in elucidating its underlying mechanisms, the pathophysiology of depression remains complex and multifactorial, involving pathways such as neuroinflammation, oxidative stress, neuroendocrine dysfunction, and metabolic disturbances (5–7). Among these factors, lipid metabolism disorders have garnered increasing attention, as dyslipidemia has been implicated as a potential contributor to depression risk (8, 9). Nevertheless, existing findings remain inconsistent, with studies reporting conflicting results regarding the strength and direction of this association (10–12).

The atherogenic index of plasma (AIP), calculated as the logarithmic transformation of the triglyceride (TG) to high-density lipoprotein cholesterol (HDL-C) ratio [log(TG/HDL-C)], has emerged as a robust marker reflecting both lipid metabolic dysfunction and cardiovascular risk profiles (13, 14). Compared with conventional lipid indices, the AIP offers a more comprehensive evaluation by capturing the balance between pro-atherogenic and anti-atherogenic lipoprotein levels. Notably, elevated AIP values have been associated with systemic inflammation, endothelial dysfunction, and insulin resistance—biological processes that may also contribute to the development of depression (15).

Recently, epidemiological investigations have begun to explore the potential relationship between the AIP and depression, particularly among subpopulations such as individuals with metabolic syndrome, cardiovascular disorders, or obesity (16). Mechanistically, dysregulated lipid metabolism may influence neural structure and function through inflammatory and oxidative pathways, thereby playing a role in affective mood disorder (17, 18). However, the current body of evidence remains fragmented, and the strength and consistency of this association are uncertain due to methodological heterogeneity across studies, including variations in study design, sample characteristics, and analytical approaches (19, 20).

Given these limitations, a meta-analysis is necessary to systematically synthesize the existing literature and provide a quantitative assessment of the association between the AIP and depression risk. This analysis may help clarify the potential role of lipid metabolic dysregulation in depressive disorders and inform strategies for early identification, risk stratification, and targeted intervention in at-risk populations.

## Methods

This meta-analysis was conducted in accordance with the guidelines outlined in the Cochrane Handbook for Systematic Reviews of Interventions and the Preferred Reporting Items for Systematic Reviews and Meta-Analysis (PRISMA) guidelines (21, 22). The study protocol was registered with PROSPERO under the registration code CRD420251035701.

### Literature search

To comprehensively identify studies relevant to the aim of this meta-analysis, a systematic search was conducted across three major databases: PubMed, Embase, and Web of Science. The search strategy included two sets of key terms: (1) “atherogenic index of plasma” OR “AIP” OR “atherogenic index” OR “lipid indices”; and (2) “depression” OR “depressive” OR “mood” OR “affective disorder” OR “depressive symptoms” OR “depressive disorder”. The search was limited to human studies and included only full-length articles published in peer-reviewed English-language journals. Additionally, the reference lists of relevant original and review articles were manually screened to identify any additional eligible studies. The search covered literature from the inception of each database through April 5, 2025.

### Inclusion criteria

Studies were included in this meta-analysis if they met the following criteria: (1) involved adult populations (aged 18 years or older), without specifically excluding individuals with pre-existing cardiovascular diseases or other chronic conditions; (2) measured AIP using the formula log(TG/HDL-C) or reported TG and HDL-C levels; (3) compared individuals with varying levels of AIP (e.g., high vs. low) or assessed AIP as a continuous variable; (4) reported the incidence or prevalence of depression in relation to AIP; (5) employed an observational study design, including cross-sectional, case-control, or cohort study designs; and (6) were published as full-length articles in peer-reviewed English-language journals.

### Exclusion criteria

Studies were excluded if they met any of the following criteria: (1) involved children or adolescents less than 18 years of age; (2) focused on patients with specific diseases rather than a general population; (3) did not measure AIP, used alternative lipid markers without calculating AIP, or reported insufficient data for calculation of AIP; (4) lacked a comparison group or did not assess the association between AIP and depression; (5) did not report on depression or used non-validated measures to assess depressive symptoms; or (6) were reviews, editorials, preclinical studies, or studies published only as abstracts.

### Study selection and data extraction

Two independent reviewers performed the study selection and data extraction using a pre-defined standardized form. For studies with unclear methodological details, the reviewers contacted the original authors to obtain additional information. Any disagreements between the reviewers were resolved through discussion or, if necessary, by consulting with a third senior investigator to reach a consensus. Extracted data included the first author’s name, publication year, country or region, participant age, sex distribution, study design, total sample size, methods used to assess depression, number of participants diagnosed with depression, and covariates for which adjustment was made in the analysis of the association between the AIP and depression.

### Quality assessment

The quality of the included studies was assessed using the Newcastle–Ottawa Scale (NOS) (23, 24), which evaluates methodological quality across three domains: selection, comparability, and outcome (or exposure). Studies scoring more than 6 out of a maximum of 9 points were considered high quality. Two reviewers independently conducted the quality, and any discrepancies were resolved by a third reviewer.

### Statistical analysis

Statistical analysis was performed according to the Cochrane Collaboration guidelines (25). Association between AIP and depression was expressed as odds ratio (OR) with 95% confidence interval (CI). ORs and standard errors (SEs) were derived from reported CIs or *p*-values and log-transformed to stabilize variance and normalize distributions. For continuous outcomes, mean differences (MDs) with corresponding 95% CIs were calculated to compare AIP values between patients and controls. Heterogeneity was assessed using the Cochrane Q test and I² statistic, with an I² value greater than 50% indicating significant heterogeneity (26). A fixed-effects model was employed if no statistical heterogeneity (*P* > 0.1, *I²* < 50%) was observed; otherwise, a random-effects model was utilized. Subgroup analyses explored the impact of study characteristics on outcomes. Publication bias was evaluated using the funnel plot and Egger’s test (27). All statistical analyses were performed using RevMan software (version 5.4, Cochrane Collaboration, Oxford, UK) and Stata software (version 14.0, Stata Corporation, College Station, TX), with *P*-values < 0.05 considered statistically significant.

## Results

### Basic characteristics and quality assessment

**Figure 1:**
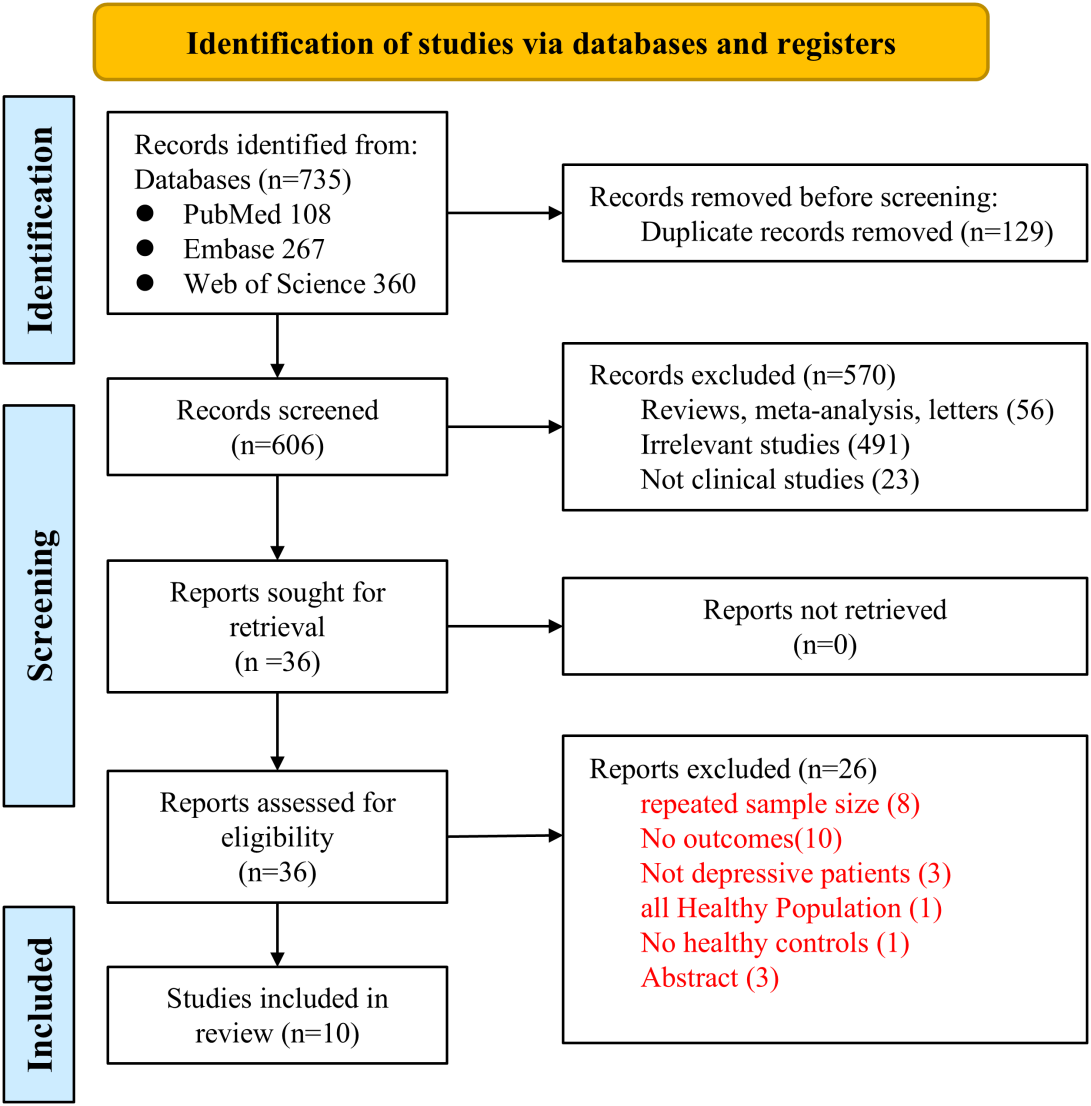
Flowchart of the study selection process.

The PRISMA flowchart is presented in **Figure 1**. Our initial literature search identified 735 studies from the PubMed, EMBASE, and Web of Science databases. Of these, 129 were duplicates, and 56 were reviews, meta-analyses, or letters. After screening of titles and abstracts, 491 studies were deemed irrelevant, and 23 were excluded for not being clinical studies. Consequently, the full texts of the remaining 36 records were assessed independently by two authors, leading to the exclusion of 26 studies for various reasons. Finally, 10 observational studies, including 5 cross-sectional studies (28–32) and 5 case-control studies (33–37), were included for subsequent quantitative analyses. These studies were published between 2009 and 2025, originating from China (3 studies), Brazil (3 studies), and one each from Poland, Turkey, Iran, and Croatia.

The summarized characteristics of the included studies are presented in **Table 1**. Depression was diagnosed using various standardized assessment tools, including the Hamilton Depression Rating Scale (HAM-D), the Diagnostic and Statistical Manual of Mental Disorders, Fourth Edition (DSM-IV), the Hamilton Depression Rating Scale (HDRS), the Beck Depression Inventory (BDI) score, the Hospital Anxiety and Depression Scale (HADS), and the Patient Health Questionnaire-9 (PHQ-9). In all studies, depression diagnoses were made by trained healthcare professionals. A total of 38,785 participants were included, of whom 3,973 were diagnosed with depression. Eight studies employed multivariate analyses to assess the association between the AIP and depression, adjusting for potential confounding factors such as age, body mass index (BMI), smoking, gender, socioeconomic status, and comorbidities of varying severity. The characteristics and quality assessment of the included studies are summarized in **Table 2**. The NOS scores for all studies ranged from 7 to 8, indicating high methodological quality.

**Table 1:** Characteristics of included studies.

Study	Country	Design	Population	Sample size	Age (years)	Male (%)	Diagnostic criteria for depression	No. of depression cases	Variables adjusted
Kalelioğlu 2018 (28)	Turkey	Cross-sectional	Male patients with depressive, and healthy controls	76	Depression: 41.54 ± 10.29 Control: 39.02 ± 10.69	100.0%	HAM-D	35	Age, BMI, smoking status, drug medication
Łucka 2017 (33)	Poland	Case-control	Elderly inpatients aged ≥60 with unipolar depression and nondepressed controls	564	76.9 ± 8.2	16.3%	DSM-IV	282	Sex and age
Martin-Subero 2014 (29)	Brazil	Cross-sectional	Participants with major depression, bipolar disorder, and normal controls at the Londrina State University	290	18-60	NA	NA	91	BMI, age, smoking, and gender
Nunes 2015 (34)	Brazil	Case-control	Adults aged 18–65 with mood disorders and controls	331	18–65	NA	DSM-IV, HDRS	134	Disorder, BMI and gender
Oliveira 2017 (35)	Brazil	Case-control	MS patients with depression and healthy controls	291	18-65	Depression: 16.7% Control: 28.9%	HADS	42	No
Sagud 2009 (36)	Croatia	Case-control	Medication-free female patients with affective disorders and healthy controls	84	Depression: 50.1 ± 6.6 Control: 44.7 ± 12.8	0.0%	DSM-IV	34	Age, smoking, menopause
Shangguan 2025 (30)	China	Cross-sectional	NHANES data collected between 1999 and 2018	28932	48.0 ± 18.7	50.3%	PHQ-9	2503	Gender, age, race, hyperlipidemia, drinking, BMI, smoking, moderate physical activities, CHD, stroke, diabetes, CKD, PIR, hypertension, education level, marital status, HbA1c, and cancer
Tao 2024 (31)	China	Cross-sectional	NHANES data collected between 2005 and 2018	7,951	60.00 (50.00-69.00)	49.2%	PHQ-9	672	Gender, age, race, education level, marital status, FIPR, BMI, diabetes, hypertension, hyperlipidemia, CVD, CKD, FPG
Tavakoli 2017 (32)	Iran	Cross-sectional	BDI score diagnosed as depression and healthy male student	100	24.2 ± 2.5	100.0%	BDI	70	Age and BMI
Yang 2022 (37)	China	Case-control	First-diagnosed drug-naïve depression patients and healthy controls	166	Depression: 27.56 ± 8.34 Control: 29.36 ± 8.64	Depression: 31.8% Control: 46.4%	DSM-IV	110	No

NA, Not available; HAM-D, Hamilton Depression Rating Scale; DSM-IV Diagnostic and Statistical Manual of Mental Disorders, Fourth Edition; HDRS Hamilton Depression Rating Scale; HADS Hospital Anxiety and Depression Scale; BDI Beck Depression Inventory; PHQ-9 Patient Health Questionnaire-9; BMI body mass index; CVD cardiovascular disease; CKD chronic kidney disease; FPG fasting plasma glucose; FIPR, family income to poverty.

**Table 2:** Newcastle–Ottawa score for risk-of-bias assessment of included studies.

Study	Population				Comparability	Outcome			Score	Evaluation
	1	2	3	4		1	2	3		
Kalelioğlu 2018 (28)	1	1	1	1	2	1	1	0	8	Good
Łucka 2017 (33)	1	1	1	1	2	1	1	0	8	Good
Martin-Subero 2014 (29)	1	1	1	1	2	1	1	0	8	Good
Nunes 2015 (34)	1	1	1	1	2	1	1	0	8	Good
Oliveira 2017 (35)	1	1	1	1	2	1	1	0	8	Good
Sagud 2009 (36)	1	0	1	1	2	1	1	0	7	Good
Shangguan 2025 (30)	1	1	1	1	2	1	1	0	8	Good
Tao 2024 (31)	1	1	1	1	2	1	1	0	8	Good
Tavakoli 2017 (32)	1	1	1	1	2	1	1	0	8	Good
Yang 2022 (32)	1	1	1	1	2	1	1	0	8	Good

### Sensitivity analysis

**Figure 2:**
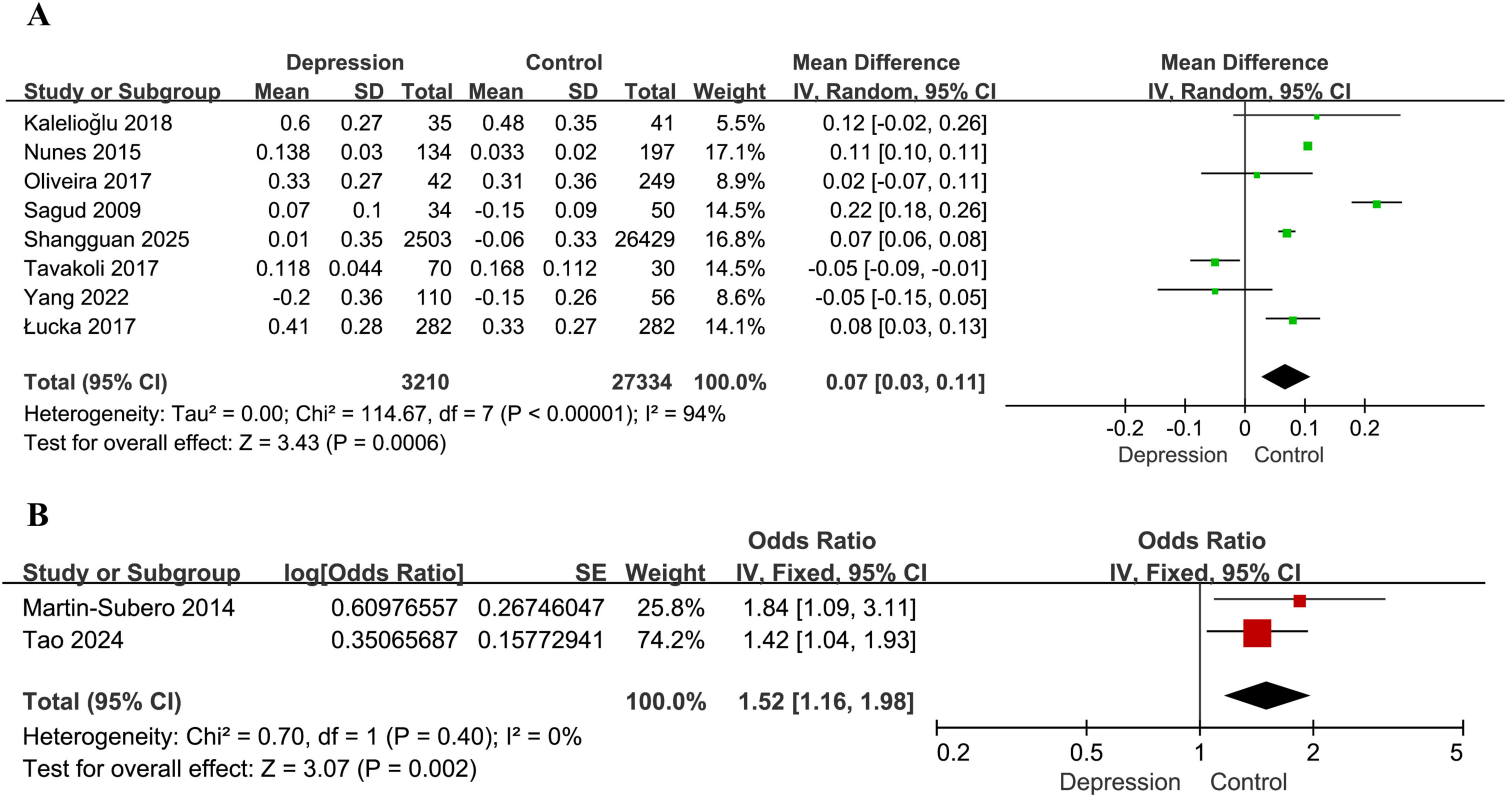
Forest plots for the meta-analysis of the association between the AIP and depression. **(A)** Forest plot of mean differences; **(B)** forest plot of odds ratios.

A total of eight studies were included in the meta-analysis examining the association between the AIP and the risk of depression (28, 30, 32–37). Among these, five studies (28, 30, 32–34) reported AIP values directly, while for the remaining three (35–37), we calculated AIP values based on the levels of TG and HDL-C. The pooled analysis demonstrated a statistically significant association between elevated AIP levels and an increased risk of depression (MD = 0.07, 95% CI: 0.03–0.11, *P* = 0.0006). However, substantial heterogeneity was observed across studies (*I²* = 94%, *P* < 0.00001; **Figure 2A**). Sensitivity analysis was performed by systematically excluding each study individually and showed that the results remained statistically significant in all scenarios (*P* < 0.05), indicating the robustness of the overall findings. Nevertheless, heterogeneity persisted at high levels (*I²* > 75%) across all sensitivity analyses, suggesting that the variability among studies could not be attributed to any single study.

Two additional studies assessed the association between the AIP and depression risk based on ORs (29, 31). Analysis using a fixed-effects model demonstrated that higher AIP values were significantly associated with depression (OR = 1.52, 95% CI: 1.16–1.98, *P* = 0.002), with no evidence of heterogeneity (*I²* = 0%, *P* = 0.40; **Figure 2B**).

### Subgroup analyses

**Figure 3:**
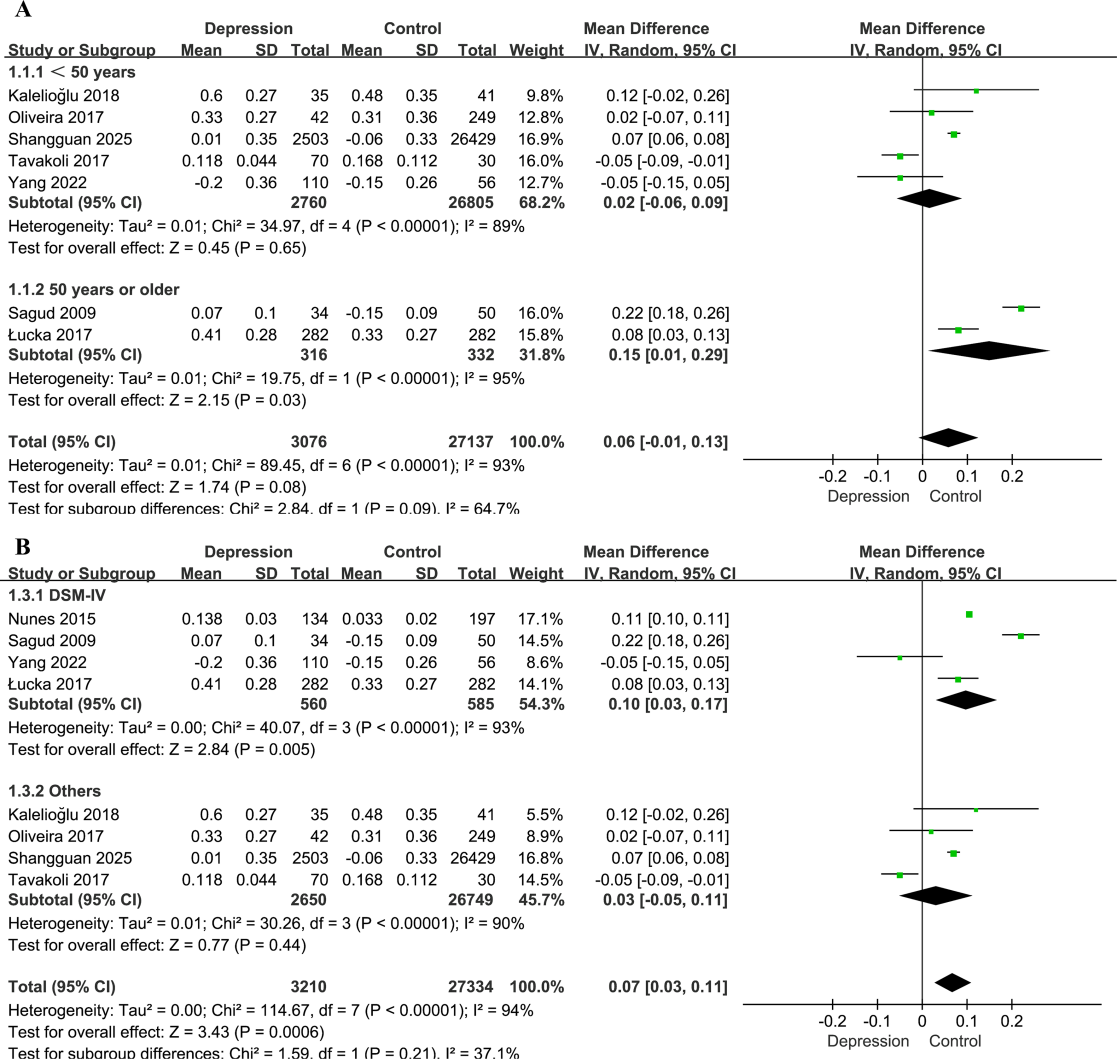
Forest plots for the subgroup analyses of the association between the AIP and depression. **(A)** Subgroup analysis according to age; **(B)** subgroup analysis according to diagnostic method for depression.

Subgroup analysis based on age (<50 vs. ≥50 years) suggested a potential modifying effect. In participants aged <50 years (28, 30, 32, 35, 37), the difference in the AIP between individuals with depression and controls was not statistically significant (MD = 0.02, 95% CI: -0.06–0.09, *P* = 0.65, *I²* = 89%). In contrast, among those aged ≥50 years (33, 36), the association was significant (MD = 0.15, 95% CI: 0.01–0.29, *P* = 0.03, *I²* = 95%). The interaction between subgroups was marginally significant (interaction *P* = 0.09, *I²* = 64.7%; **Figure 3A**). One study reporting only median age was excluded from this analysis.

Studies employing the DSM-IV diagnostic criteria (33, 34, 36, 37) showed a stronger association (MD = 0.10, 95% CI: 0.03–0.17, *P* = 0.005) than those using other diagnostic standards (28, 30, 32, 35) (MD = 0.03, 95% CI: −0.05–0.11, *P* = 0.44). However, the difference between subgroups was not statistically significant (*χ²* = 1.59, *P* = 0.21, I² = 37.1%; **Figure 3B**).

### Publication bias

**Figure 4:**
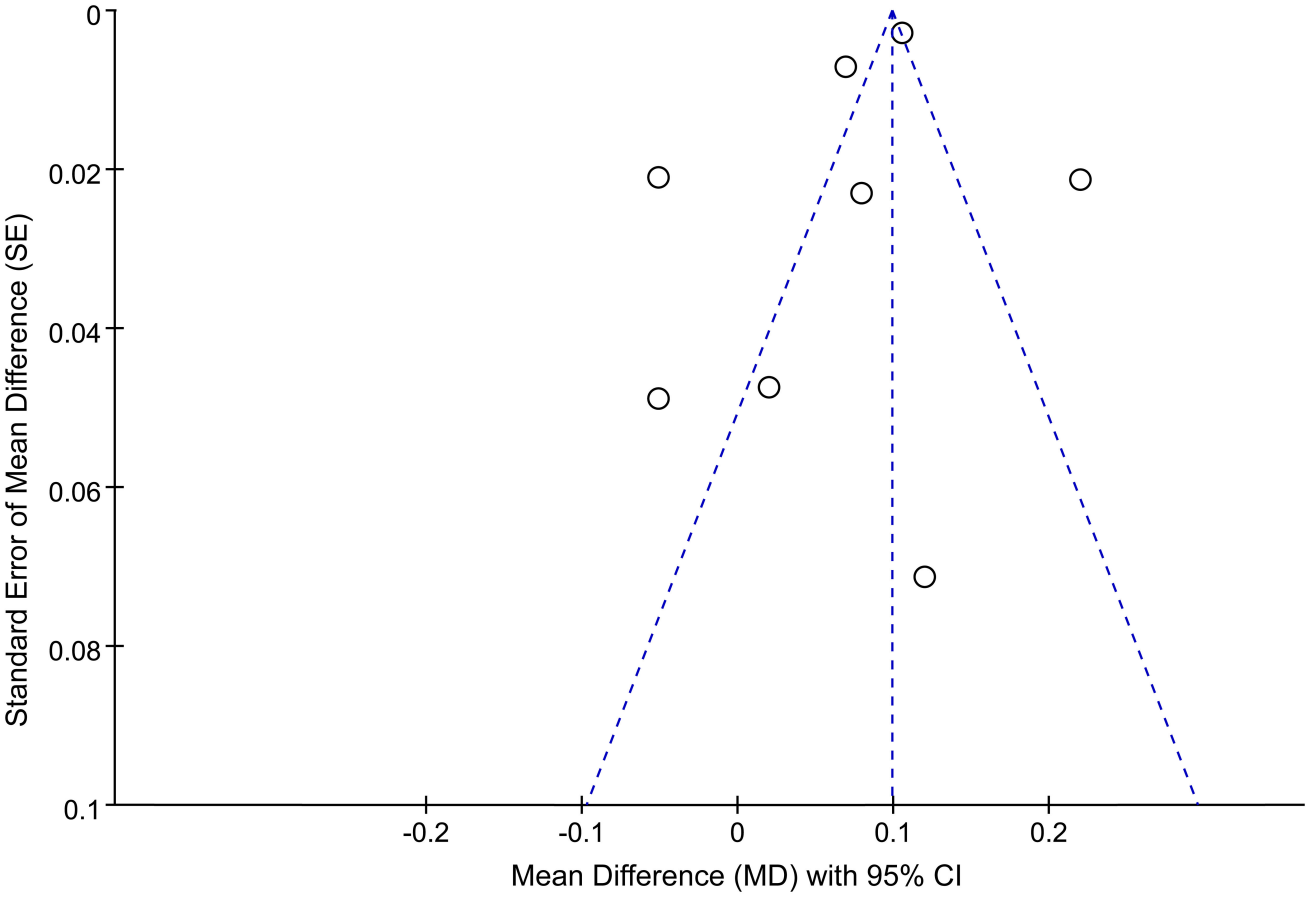
Funnel plots for publication bias among studies of the association between the AIP and depression.

**Figure 5:**
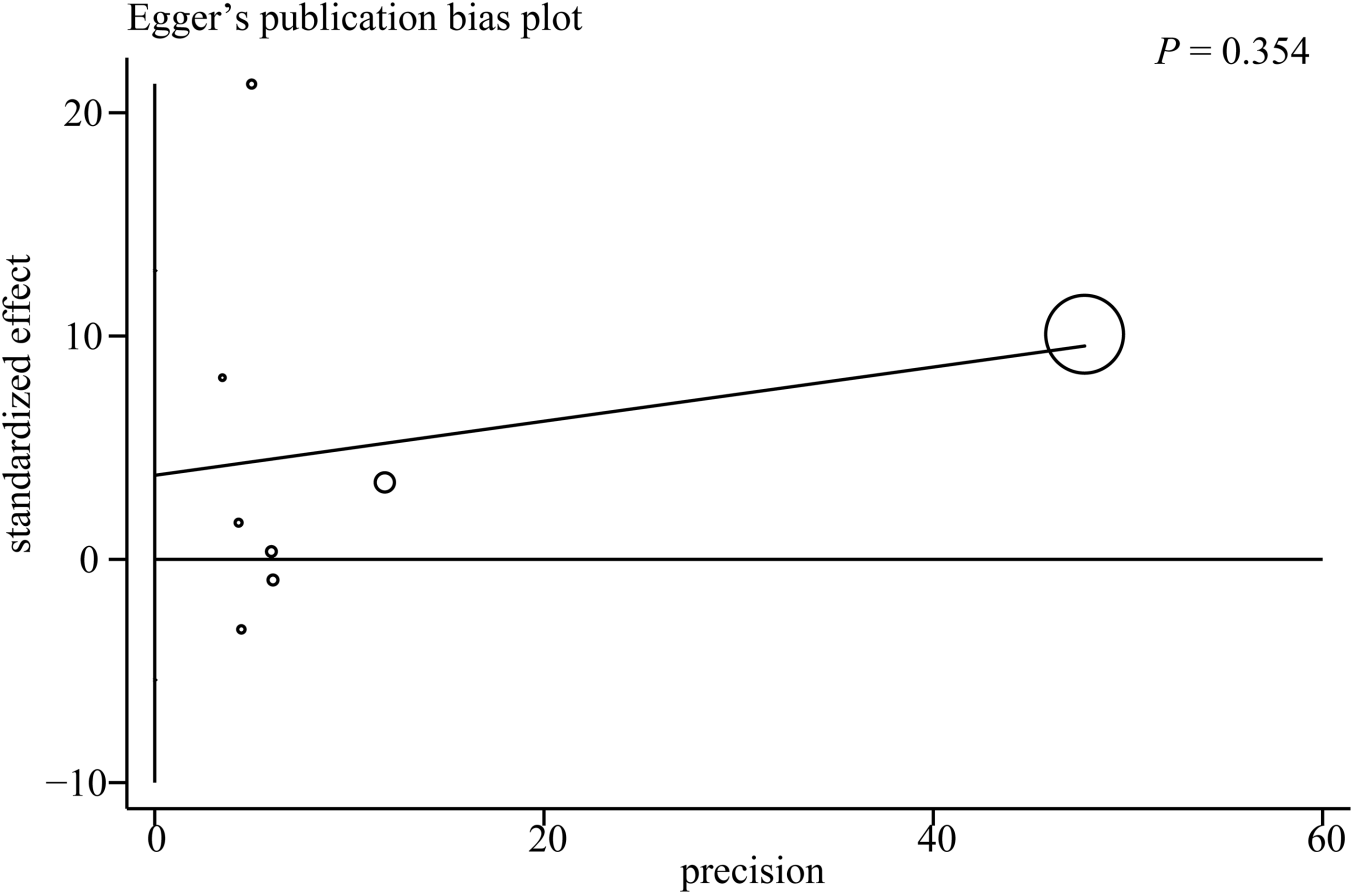
Egger’s test results for the association between the AIP and depression.

Publication bias was assessed using a funnel plot and Egger’s test. The funnel plot evaluating the association between the AIP and depression risk is presented in **Figure 4**. Four studies were located outside the funnel boundaries, suggesting potential publication bias or heterogeneity. However, Egger’s test indicated no statistically significant publication bias (*P* = 0.354; **Figure 5**).

## Discussion

This meta-analysis, which included 10 observational studies, encompassing 38,785 participants with 3,973 cases of depression, demonstrated a significant positive association between higher AIP values and an increased risk of depression (MD = 0.07, 95% CI: 0.03–0.11, *P* = 0.0006). Given that the AIP is a simple, readily accessible measure based on TG and HDL-C levels, these findings suggest that the AIP could serve as a potential risk indicator for depression, especially in individuals aged 50 years and older. This association is especially relevant in clinical settings, where the AIP could provide added value in identifying individuals at elevated risk for mood disorders.

The biological credibility of this association is underlined by multiple interconnected pathophysiological processes. An elevated AIP reflects an imbalance in lipid metabolism, commonly associated with insulin resistance, chronic inflammation, and oxidative stress, all of which have been implicated in the pathogenesis of depression (38, 39). In particular, insulin resistance has been shown to influence central nervous system function by modulating inflammatory cytokine levels, neurotransmitter metabolism, and neuroplasticity (40, 41). These mechanisms may lead to impaired synaptic function and reduced hippocampal neurogenesis, both of which are key features observed in major depressive disorder (42, 43). In addition, individuals with elevated AIP values often exhibit systemic inflammation, which may compromise the blood–brain barrier. This compromise facilitates the passage of proinflammatory cytokines into the brain, where they may interfere with neurochemical circuits that regulate affective processes (44, 45). Inflammatory markers such as interleukin-6 (IL-6) and tumor necrosis factor-alpha (TNF-α) are consistently elevated in patients with depression (46). These cytokines are also known to influence the tryptophan–kynurenine metabolic pathway, shifting tryptophan utilization away from serotonin biosynthesis toward the formation of neurotoxic intermediates, potentially exacerbating depressive symptoms (47).

The AIP has been identified as a surrogate marker for metabolic syndrome (MS), which comprises a constellation of metabolic abnormalities such as central obesity, elevated blood pressure, impaired glucose metabolism, and dyslipidemia (48). Several meta-analyses have demonstrated a significant link between MS and depressive disorders, indicating that disturbances in metabolic and emotional regulation may arise via shared pathophysiological mechanisms (49, 50). In this context, the AIP functions not only as an indicator of lipid dysregulation but also as a potential comprehensive marker for systemic metabolic dysfunction. Furthermore, increased AIP values have been correlated with an elevated risk of cardiovascular events (51). The reciprocal relationship between cardiovascular disease (CVD) and depression is well substantiated (52). Depression is both a risk factor for and a consequence of CVD, and this interaction may further magnify the clinical implications of an elevated AIP (53).

Our subgroup analysis showed that the association between the AIP and depression was more evident in individuals aged 50 years and older. This observation is consistent with prior studies involving metabolic indicators such as the TG–glucose (TyG) index, which have reported stronger links to depression among middle-aged and elderly groups (54, 55). Factors such as diminished metabolic adaptability, heightened systemic inflammation, and age-related vascular changes may intensify the influence of lipid disturbances on mental health in older adults (56, 57). These age-related patterns further suggest that the AIP could serve as a valuable marker for evaluating metabolic and psychiatric risk specifically within geriatric populations (58).

Several limitations of the present study should be considered. First, all included studies had either cross-sectional or case-control designs, limiting the ability to establish causality. Prospective cohort studies are needed to confirm the directionality of the association. Second, substantial heterogeneity was observed among the studies (*I²* = 94%, *P* < 0.00001), likely attributable to differences in study populations, diagnostic tools for depression, and variations in AIP calculation methods. Third, although most studies adjusted for key confounders such as age, sex, and BMI, residual confounding by factors like diet, physical activity, medication use, management of comorbidities, and socioeconomic status cannot be excluded. Fourth, variation in depression assessment methods (e.g., different diagnostic criteria or scales) precluded subgroup analysis by depression severity. Future studies should consider using standardized criteria and exploring whether associations differ by severity, as suggested by Medhi et al. (59).

Despite extensive literature on cardiovascular risk, metabolic disorders, and depression, the present study provides novel and complementary insights by demonstrating the value of the AIP as an integrative lipid biomarker that reflects the balance between atherogenic and protective lipoproteins and thus offers more predictive potential than single lipid measures. To our knowledge, this is the first meta-analysis to quantify the overall association between AIP and depression risk, and thereby reveal its potential as a clinically relevant biomarker.

Future research should prioritize large-scale, prospective studies to assess whether elevated AIP values independently predict depression, alongside mechanistic investigations into the biological pathways linking dyslipidemia to mood disorders—focusing on inflammation, oxidative stress, MS, and cardiovascular health. Interventional studies evaluating the impact of lifestyle or pharmacological strategies to reduce the AIP on depression risk would also offer valuable clinical insights. From a clinical standpoint, routine AIP monitoring, especially in older adults, could aid in the early identification of high-risk individuals, and integrating metabolic and mental health assessments may enhance prevention, risk stratification, and personalized intervention strategies.

## Conclusion

In summary, this meta-analysis reveals a significant association between higher AIP values and an increased risk of depression, particularly among individuals aged 50 years and older. The use of AIP, as an integrated lipid marker, provides a novel perspective beyond conventional lipid parameters, and this study is the first to quantify this pooled association. Despite substantial heterogeneity across the included studies, the association remained stable in sensitivity analyses, and Egger’s test indicated no evidence of significant publication bias. Future research should focus on clarifying the causal relationship between the AIP and depression and exploring AIP-targeted interventions as potential preventive strategies against depressive disorders.

## Data Availability

The original contributions presented in the study are included in the article/supplementary material. Further inquiries can be directed to the corresponding authors.
